# The first report of polymorphisms of the prion protein gene (*PRNP*) in Pekin ducks (*Anas platyrhynchos domestica*)

**DOI:** 10.3389/fvets.2023.1273050

**Published:** 2023-11-08

**Authors:** Min-Ju Jeong, Zerui Wang, Wen-Quan Zou, Yong-Chan Kim, Byung-Hoon Jeong

**Affiliations:** ^1^Korea Zoonosis Research Institute, Jeonbuk National University, Iksan, Republic of Korea; ^2^Department of Bioactive Material Sciences, Jeonbuk National University, Jeonju, Republic of Korea; ^3^Department of Pathology, Case Western Reserve University School of Medicine, Cleveland, OH, United States; ^4^Department of Biological Sciences, Andong National University, Andong, Republic of Korea

**Keywords:** duck, prion protein gene, *PRNP*, polymorphism, Pekin duck, susceptibility

## Abstract

**Background:**

Prion diseases have been extensively reported in various mammalian species and are caused by a pathogenic prion protein (PrP^Sc^), which is a misfolded version of cellular prion protein (PrP^C^). Notably, no cases of prion disease have been reported in birds. Single nucleotide polymorphisms (SNPs) of the prion protein gene (*PRNP*) that encodes PrP have been associated with susceptibility to prion diseases in several species. However, no studies on *PRNP* polymorphisms in domestic ducks have been reported thus far.

**Method:**

To investigate *PRNP* polymorphisms in domestic ducks, we isolated genomic DNA from 214 Pekin duck samples and sequenced the coding region of the Pekin duck *PRNP* gene. We analyzed genotype, allele, and haplotype distributions and linkage disequilibrium (LD) among the SNPs of the Pekin duck *PRNP* gene. In addition, we evaluated the effects of the one non-synonymous SNP on the function and structure of PrP using the PROVEAN, PANTHER, SNPs & GO, SODA, and AMYCO *in silico* prediction programs.

**Results:**

We found five novel SNPs, c.441 T > C, c.495 T > C, c.582A > G, c.710C > T(P237L), and c.729C > T, in the ORF region of the *PRNP* gene in 214 Pekin duck samples. We observed strong LD between c.441 T > C and c.582A > G (0.479), and interestingly, the link between c.495 T > C and c.729C > T was in perfect LD, with an *r*^2^ value of 1.0. In addition, we identified the five major haplotype frequencies: TTACC, CTGCC, CTACC, CCGCT, and CTATC. Furthermore, we found that the non-synonymous SNP, c.710C > T (P237L), had no detrimental effects on the function or structure of Pekin duck PrP. However, the non-synonymous SNP had deleterious effects on the aggregation propensity and solubility of Pekin duck PrP compared with wildtype Pekin duck PrP.

**Conclusion:**

To the best of our knowledge, this study is the first report on the genetic characteristics of *PRNP* SNPs in Pekin ducks.

## Introduction

Prion diseases are caused by an accumulation of pathogenic prion protein (PrP^Sc^) in the brain and are fatal neurodegenerative diseases that are also called transmissible spongiform encephalopathies (TSEs) (1, 2). PrP^Sc^ is derived from the conversion of normal cellular prion protein (PrP^C^) via protein misfolding and is characterized by a rich-β-sheet structure and insolubility in detergent (2). Prion diseases occur in a broad range of host species, including Creutzfeldt–Jakob disease (CJD) in humans, scrapie in sheep and goats, bovine spongiform encephalopathy (BSE) in cattle, feline spongiform encephalopathy (FSE) in cats, and camel prion disease (CPD) (3–5). Interestingly, no cases of prion disease infection have been reported in birds up to date.

The prion protein gene (*PRNP*), which encode the prion protein (PrP), is a key molecule in prion diseases (1–3). Notably, single nucleotide polymorphisms (SNPs) of the *PRNP* gene have been reported to be associated with susceptibility to prion diseases across several species. In humans, the Met homozygote at codon 129 of the *PRNP* gene contributes to susceptibility to sporadic and variant CJD (1, 6–10). In addition, haplotypes formed by codons 136, 154, and 171 within the ovine *PRNP* gene have been classified into five risk groups based on the degree of susceptibility to scrapie in sheep (3, 11, 12). Similarly, caprine *PRNP* SNPs at codons 102, 127, 142, 143, 146, 154, 211, and 222 have been used to assess the vulnerability of goats to scrapie (11, 13–27). It’s worth noting that no polymorphisms related to this disease have been detected in the *PRNP* gene in dromedaries (28).

Among several animals described as the prion disease-resistant species, chickens showed strong resistance to prion diseases. During the BSE outbreak in the United Kingdom, which resulted from the consumption of prion-contaminated animal products containing meat- and bonemeal, widespread instances of prion disease transmission from humans to domestic cats were reported (29), but no cases of prion disease infection in chickens have been reported thus far. In addition, exposing chickens to parenteral or oral doses of BSE agent to did not result in prion disease transmission (30). Remarkably, although many SNPs associated with susceptibility to prion disease have been reported in prion disease–sensitive species, no SNPs were detected in four breeds of the large-scale chicken population: Korean native chicken, Ogolgye, Ross, or Dekalb White (31, 32).

Similar to chickens, ducks are widely bred and consumed around the world. In a previous study, the characteristics of the *PRNP* sequence in domestic ducks were analyzed in various perspectives (33). Notably, domestic duck PrP showed a higher proportion of β-sheet structure and propensity for aggregation compared with chicken PrP (33). In addition, when a substitution to incorporate a specific amino acid from chicken PrP occurred in the PrP sequence of domestic ducks, the amyloid propensity was observed to decrease, compared with that of wildtype duck (33). Furthermore, polymorphisms of the *PRNP* gene have been identified in many animals (10, 31, 32, 34–44). However, no studies on *PRNP* polymorphisms in domestic ducks have yet been reported.

Therefore, in this study, we investigated the genotype and allele frequencies of *PRNP* polymorphisms in a group of 214 Pekin ducks and analyzed the haplotype distribution and linkage disequilibrium (LD) of the polymorphisms we found. In addition, we compared the distribution of polymorphisms in the open reading frame (ORF) of the *PRNP* gene between chickens and Pekin ducks. Furthermore, we evaluated the effects of the lone non-synonymous SNP on the molecular characteristics of PrP using PROVEAN, PANTHER, SNPs & GO, SODA, and AMYCO *in silico* prediction programs.

## Materials and methods

### Sample preparation

All 214 samples from Pekin ducks were obtained from a slaughterhouse located in the Republic of Korea. The Labopass Tissue Genomic DNA Isolation Kit (Cosmo Genetech Co., Ltd., Korea) was used to isolate genomic DNA from 20 mg of peripheral tissue by following the manufacturer’s manuals. The overall experimental protocols were approved by the Institutional Animal Care and Use Committee of Jeonbuk National University (CBNU 2017-0030). All experiments using Pekin ducks were performed in accordance with the Korean Experimental Animal Protection Act.

### Genetic analysis of the Pekin duck *PRNP*

In previous studies (33), a pair of primers was designed based on the *PRNP* sequence of the mallard (*Anas platyrhynchos*), which is available in GenBank at the National Center for Biotechnology Information (Gene ID: AF283319.1). The gene-specific sense and anti-sense primers are as follows: TGGTGCAGACAACAGCTGGG and TGGGCTCAGGGACACGAAGA, respectively. Using these primers, polymerase chain reaction (PCR) targeting the coding region of Pekin duck *PRNP* gene was performed on an S-1000 Thermal Cycler (Bio-Rad, Hercules, CA, United States). The PCR conditions followed the manual for BioFACT^™^ Taq DNA Polymerase (BioFACT Co., Ltd., Daejeon, Korea) with an annealing temperature of 65°C. The amplified products were purified using a FavorPrep^™^ GEL/PCR Purification Kit (Favorgen Biotech Corp., Kaohsiung, Taiwan), and sequencing was carried out on an ABI PRISM 3730XL Analyzer (ABI, Foster City, CA, United States). The obtained sequencing results for each sample were analyzed using Finch TV software (Geospiza Inc., Seattle, WA, United States), and subsequent genotyping was performed.

### *In silico* prediction of the effects of the non-synonymous SNPs

PROVEAN, PANTHER, and SNP&GO are *in silico* analysis tools used to assess the effect of non-synonymous SNPs on the structure or function of a protein. PROVEAN evaluates the effect of non-synonymous SNPs by building and comparing clusters of related sequences and predicting the score. The results classify SNPs as “deleterious” or “neutral,” according to a predefined threshold (e.g., −2.5). PANTHER estimates the effect of non-synonymous SNPs using PANTHER-PSEP (position-specific evolutionary preservation) and denotes the results as “probably damaging,” “possibly damaging” and “probably benign” (45). SNP&GO predicts the effects of non-synonymous SNPs by categorizing the functional and evolutional information about a protein sequence with a support vector machine and labels the results as “disease” or “neutral.” Missense3D predicts structural changes caused by deleterious damage to protein stability resulting from missense variants. It identifies structural damage through a detailed analysis that includes the examination of various factors such as the disruption of buried salt bridges and alterations in secondary structure. AMYCO is an *in silico* analysis application that predicts the propensity to aggregation into an amyloid and presents the results as visualized scores from 0 to 1. SODA is a webserver for *in silico* analyses that predicts the effects of amino acid variations on protein solubility. The SODA result is deduced by comparing the profiles, which include the propensity of a protein sequence to aggregation and intrinsic disorder, hydrophobicity, and secondary structure preferences, of reference (wildtype) and mutated sequences.

### Statistical analysis

The Hardy–Weinberg equilibrium (HWE) test was conducted using the Michael H. Court calculator. If the resulting *p*-value is greater than 0.05, the genotype frequencies are in the HWE. The haplotype distribution and LD were estimated using Haploview version 4.2 (Broad Institute, Cambridge, MA, United States). LD values were measured using the coefficient *r*^2^ range from 0 to 1, with *r*^2^ > 0.3 representing strong LD.

## Results

### Investigation of *PRNP* polymorphisms in Pekin ducks

**Figure 1:**
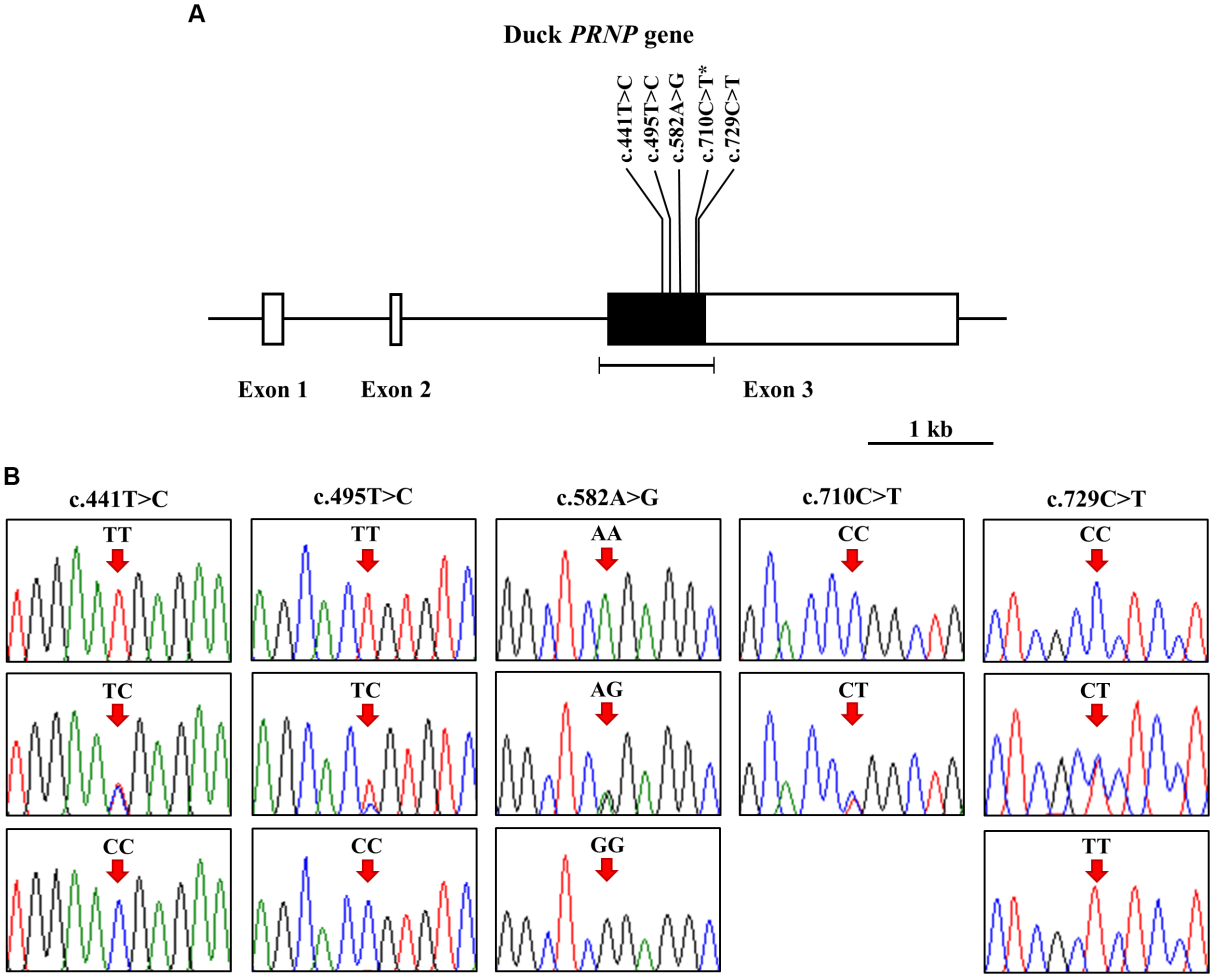
Identification of single-nucleotide polymorphisms (SNPs) in the Pekin duck prion protein gene (*PRNP*). **(A)** The schematic diagram illustrates the genomic structure of the Pekin duck *PRNP*. The open reading frame (ORF) within exon 3 is represented by the black box, and the white boxes depict the 5′ and 3′ untranslated regions (UTRs). The edged horizontal bar indicates the regions sequenced. The locations of the identified polymorphisms in this study are shown in bold, with an asterisk denoting the non-synonymous SNP. **(B)** Five novel SNPs were discovered within the ORF of the duck *PRNP*. Electropherograms display two or three genotypes at c.441 T > C, c.495 T > C, c.582A > G, c.710C > T (P237L), and c.729C > T. The homozygote of the minor allele at c.710C > T was not found. The colors of the peaks represent each base of the DNA sequence as follows: green for adenine; red for thymine; blue for cytosine; black for guanine. Arrows indicate the locations of the polymorphisms identified in this study. Upper panel, homozygote of the major allele; middle panel, heterozygote; lower panel, homozygote of the minor allele.

To investigate polymorphisms in the *PRNP* of the Pekin duck (*Anas platyrhynchos domesticus*), we performed PCR and automatic amplicon sequencing on 214 Pekin duck samples. We found five novel SNPs, c.441 T > C, c.495 T > C, c.582A > G, c.710C > T, and c.729C > T, in the ORF region of the Pekin duck *PRNP* (Figures 1A,B). Among them, only c.710C > T (P237L) was a non-synonymous SNP. Detailed information about the genotype and allele frequencies of these SNPs is provided in Table 1; Supplementary Figure S1. All SNPs were in the HWE (*p* > 0.05).

**Table 1:** Genotype and allele frequencies of prion protein gene (*PRNP*) polymorphisms in 214 Pekin ducks.

Polymorphisms	Genotype frequency, *n* (%)			Total, *n* (%)	Allele frequency, *n* (%)		Total, *n* (%)	HWE
c.441 T > C	TT	TC	CC		T	C		
(N147N)	61	102	51	214	224	204	428	0.51
	(28.5)	(47.7)	(23.8)	(100)	(52.3)	(47.7)	(100)	
c.495 T > C	TT	TC	CC		T	C		
(T165T)	175	36	3	214	386	42	428	0.47
	(81.8)	(16.8)	(1.4)	(100)	(90.2)	(9.8)	(100)	
c.582A > G	AA	AG	GG		A	G		
(S194S)	105	88	21	214	298	130	428	0.68
	(49.1)	(41.1)	(9.8)	(100)	(69.6)	(30.4)	(100)	
c.710C > T	CC	CT	TT		C	T		
(P237L)	196	18	0	214	410	18	428	0.52
	(91.6)	(8.4)	(0.0)	(100)	(95.8)	(4.2)	(100)	
c.729C > T	CC	CT	TT		C	T		
(A243A)	175	36	3	214	386	42	428	0.47
	(81.8)	(16.8)	(1.4)	(100)	(90.2)	(9.8)	(100)	

The extent of LD among the five *PRNP* SNPs was investigated using *r*^2^ values (Table 2). Strong LD (*r*^2^ > 0.3) was observed between c.441 T > C and c.582A > G (0.479), and the link between c.495 T > C and c.729C > T was in perfect LD, with an *r*^2^ value of 1.0. The remaining SNPs exhibited weak links, with *r*^2^ scores of less than 0.3. As shown in Table 3; Supplementary Figure S2, we also analyzed the haplotype frequency. The five major haplotypes were TTACC, CTGCC, CTACC, CCGCT, and CTATC, with frequencies of 52.4, 20.6, 13.1, 9.8, and 4.2%, respectively.

**Table 2:** Linkage disequilibrium (LD) of prion protein gene (*PRNP*) polymorphisms in Pekin ducks.

	c.441 T > C	c.495 T > C	c.582A > G	c.710C > T	c.729C > T
c.441 T > C	–	0.119	0.479*	0.048	0.119
c.495 T > C	–	–	0.249	0.005	1.0**
c.582A > G	–	–	–	0.019	0.249
c.710C > T	–	–	–	–	0.005
c.729C > T	–	–	–	–	–

*r^2^ > 0.3 indicates strong LD.

**r^2^ = 1 indicates perfect LD.

**Table 3:** Haplotype frequencies of prion protein gene (*PRNP*) polymorphisms in Pekin ducks.

Haplotype	Frequency, *n* (%)
TTACC	224 (52.4%)
CTGCC	88 (20.6%)
CTACC	56 (13.1%)
CCGCT	42 (9.8%)
CTATC	18 (4.2%)
Total, *n* (%)	428

### Comparison of polymorphism distributions among avian species

In previous studies, only 2 insertion/deletion polymorphisms of the chicken *PRNP* gene, c.163_180delAACCCAGGGTACCCCCAT and c.268_269insC, were found in 4 chicken breeds. Of them, c.163_180delAACCCAGGGTACCCCCAT is a hexapeptide deletion polymorphism in unit (U) 2 of the tandem repeat spanning U1 to U7 (32). Interestingly, a substantial number of polymorphisms, consisting of 33 SNPs in quails and 28 SNPs along with six insertion/deletion polymorphisms in pheasants, were identified within their *PRNP* gene (41, 42). Moreover, when compared to chickens, the structure of the hexapeptide repeat region in both quails and pheasants remains highly conserved. However, unlike in chicken, we found no insertion/deletion polymorphisms in the Pekin duck *PRNP*. In fact, we found no variations within the hexapeptide repeat region. Instead, the Pekin duck *PRNP* exhibited a non-synonymous SNP and 4 synonymous SNPs. The different distributions of *PRNP* polymorphisms among avian species are summarized in Table 4.

**Table 4:** Distribution of polymorphisms within the open reading frame (ORF) of the prion protein gene (*PRNP*) among avian species.

Species	Polymorphisms	Total, *n*	References
Chicken	c.163_180delAACCCAGGGTACCCCCAT (NPGYPH), c.268_269insC	2	Kim et al. (31, 32)
Quail	c.12C > T, c.15C > T, c.56C > T (T19I), c.60C > T, c.61G > A (V21I), c.64G > T (A22S), c.111 T > C, c.126C > T, c.144C > T, c.162 T > C, c.168G > A, c.171G > A;C, c.174 T > C, c.186A > G, c.192C > T, c.222C > T, c.321G > A, c.357G > A, c.453C > T, c.459G > A, c.463C > A, c.474C > T, c.486G > A, c.540 T > C, c.546C > T, c.645C > T, c.678G > T, c.685C > T, c.702G > A, c.705 T > C, c.714C > T, c.806A > G, c.811G > A	33	Kim et al. (41)
Pheasant	c.-6G > A, c.60C > T, c.61G > T (V21F);C (V21L), c.67C > T (L23F), c.97G > A (G33C), c.105 T > C, c.156C > T, c.163_180delAACCCGGGGTATCCCCAC, c.168G > A, c.171G > A, c.180_181insAACCCGGGGTATCCCCAC, c.180_181insAACCCGGGGTATCCCCACAACCCGGGGTATCCCCAC, c.189A > G,C, c.192 T > C, c.198_199insAACCCAGGATATCCCCAC, c.207G > C, c.210C > T, c.216C > T, c.216_217insAACCCGGCTATCCCCACAACCCCGGCTATCCCCAC, c.219C > T, c.222C > T, c.378G > A, c.405G > T, c.411C > T, c.530G > A (R177Q), c.546C > T, c.564C > T, c.624_626delGAA, c.690G > A, c.750C > G (I250M), c.766G > A (D256N), c.781G > A (V261I)	34	Kim et al. (42)
Duck	c.441 T > C, c.495 T > C, c.582A > G, c.710C > T (P237L), c.729C > T	5	This study

### Predicting the effects of the non-synonymous SNP on the function and properties of PrP

**Figure 2:**
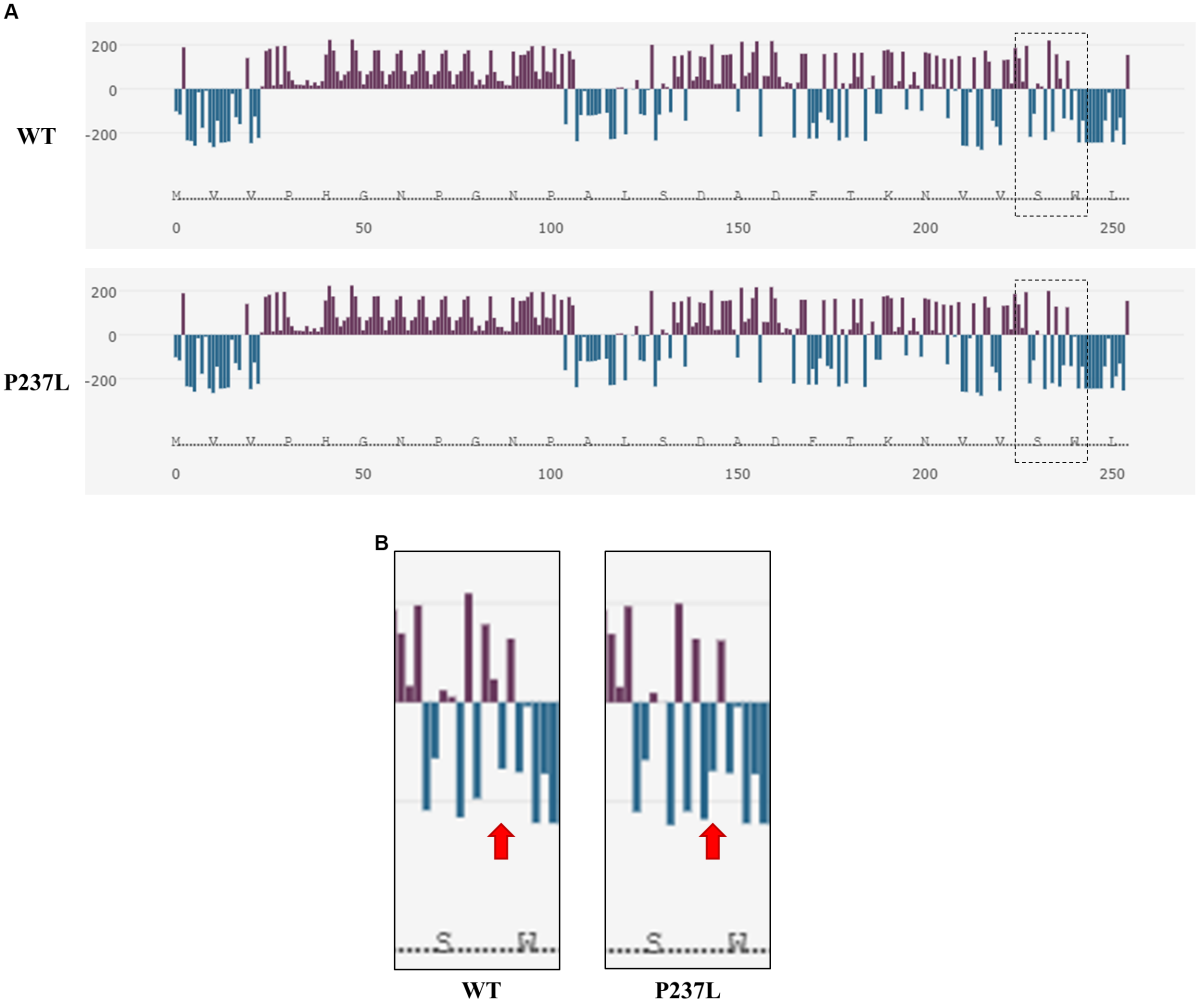
Prediction of the functional properties of the non-synonymous SNP in the Pekin duck prion protein (PrP). **(A)** The solubility analysis of Pekin duck PrP by SODA. Upper panel shows the distribution of solubility extent in the wildtype PrP sequence, and the lower panel shows the distribution of solubility extent in the PrP sequence with the non-synonymous SNP substitution (P237L). The purple bar graphs above the horizontal line indicate high solubility, and the aqua bar graphs below the horizontal line indicate low solubility. The dotted box on the right side represents the region adjacent to the non-synonymous SNP (P237L). **(B)** Enlarged view of the region inside the dotted box shown in **(A)**. The left panel displays the wildtype PrP sequence, and the right panel displays the PrP sequence with the non-synonymous SNP substitution (P237L). The arrows indicate the location of codon 237.

We evaluated the influence of the non-synonymous SNP, c.710C > T (P237L), on the Pekin duck PrP using *in silico* analysis tools. To estimate the extent to which the non-synonymous SNP had a damaging effect on the Pekin duck *PRNP*, we used PROVEAN, PANTHER, and SNPs & GO (Table 5). All three programs predicted that the c.710C > T (P237L) variant would not have a harmful effect according to their respective thresholds. We also utilized Missense3D to analyze the impact of amino acid substitutions on the PrP structure, and no structural damage was detected as a result of the non-synonymous SNP. In addition, we used AMYCO to assess the aggregation propensity of the Pekin duck PrP sequence according to the allele of the non-synonymous SNP. The PrP variant with P237L was predicted to have an increased propensity for aggregation, with a score of 0.44 (Table 5). We used the SODA program to estimate protein solubility according to the amino acid substitutions that result from the c.710C > T (P237L) SNP. SODA predicted that the P237L variant would exhibit reduced solubility compared with the reference sequence (wildtype), with a SODA score of −26.439 (Table 5). The extent of solubility in wildtype PrP and PrP with the P237L allele is described in Figure 2A. As predicted, PrP with the P237L allele was less soluble than wildtype PrP (Figure 2B).

**Table 5:** Prediction of functional effects and aggregation propensity of the non-synonymous single nucleotide polymorphisms in the Pekin duck prion protein gene (*PRNP*) on PrP.

Variation	Method	Score	Prediction
c.710C > T (P237L)	PROVEAN	−0.011^a^	Neutral
	PANTHER	2	Probably benign
	SNPs & GO	0.068^b^	Neutral
	AMYCO	0.44	Aggregation-prone
	SODA	−26.439	Less soluble

a PROVEAN prediction cutoff = −2.5.

b Disease probability, >0.5 mutation is classified as “Disease”.

## Discussion

Previous studies have reported that the PrP and *PRNP* of prion-resistant animals, including dogs, horses, and chickens, have unique genetic characteristics compared with prion-susceptible animals. An aspartic acid (Asp) at codon 163 on canine PrP has been reported to contribute to its resistance properties by stabilizing the protein structure compared with the amino acid found there in susceptible animals (46, 47). An Asp at codon 167 in horses has been reported to be involved in the β2–α2 loop, which maintains the well-defined PrP structure and contributes to its disease resistance (48–51).

Chickens are characterized by a different tandem repeat structure in *PRNP* compared with mammals, and the resulting hexapeptide structures appear to contribute to the structural stability of PrP in chickens (31, 52, 53). In addition, no SNPs have been reported in chickens (31, 32), which is a distinct difference from prion disease–sensitive animals, for which many SNPs have been reported. Interestingly, an amino acid substitution resulting from a hexapeptide deletion polymorphism (c.163_180delAACCCAGGGTACCCCCAT), one of two insertion/deletion polymorphisms found in chickens, was found to have a detrimental effect on protein function (31).

A recent study reported that the sequence of *PRNP* in domestic ducks (Pekin duck) and wildtype ducks (mallard) shows low similarity to that in chickens (33). Pekin duck PrP has a higher proportion of β-sheet structure, which is a characteristic of PrP^Sc^, and it is predicted to have a higher propensity for aggregation than chicken PrP (33). Interestingly, replacing amino acids residues in the Pekin duck PrP sequence (codons 165 and 167) with those found in chicken PrP has been found to reduce the propensity for PrP aggregation. Thus, nucleotide changes could result in disease-resistant alterations of an amino acid sequence that tends to be susceptible to prion diseases.

In this study, we first investigated polymorphism in the *PRNP* of domestic Pekin ducks. We found five novel SNPs within the ORF region of the *PRNP* gene in Pekin ducks. This is in contrast to chickens, in which no SNPs were detected (Table 4). However, it is unclear whether the rare non-synonymous SNP observed in Pekin ducks are unique to this breed or are common among domestic ducks. Thus, further research on the genetic polymorphisms of the *PRNP* gene in other duck breeds is highly desirable in the future.

Among the five SNPs found in Pekin ducks, the only non-synonymous SNP (c.710C > T, P237L) presents two interesting amino acids when compared with the *PRNP* sequences of 10 species. P237 is the wildtype amino acid in the PrP sequence of Pekin ducks and is identical to the amino acid located at the same position in chickens, mallards, and geese. L237, on the other hand, is a specific amino acid found only in the sequence of Pekin duck PrP.

Furthermore, we assessed the effect of the non-synonymous SNP (P237L) on Pekin duck PrP using *in silico* estimation tools (Table 5). PROVEAN, PANTHER, and SNPs & GO predicted that P237L would have no detrimental effect on the function or structure of the protein. AMYCO predicted that the Pekin duck PrP sequence with a Leu at codon 237 would be aggregation-prone (0.44); however, the wildtype sequence of Pekin duck PrP was also predicted to have the same score in a previous study (33). Thus, P237L seems to have no significant effect on the aggregation propensity of Pekin duck PrP. However, the amino acid substitution has a prominent effect on protein solubility. SODA predicted that the Pekin duck PrP sequence with a Leu at codon 237 would be less soluble than wildtype Pekin duck PrP, returning a score of −26.439. We confirmed that the solubility propensity of the surrounding amino acids was altered by an amino acid change at codon 237 (Figure 2).

Domestic ducks are popularly consumed around the world, and internal organs such as the brain, heart, kidney, and liver are also commonly eaten. A previous study reported the presence of amyloids in commercially available duck- and goose-derived foie gras, even after cooking (54). Furthermore, when mice were inoculated with amyloid-containing foie gras, amyloid deposition was observed in several organs Furthermore, when mice were inoculated with amyloid-containing foie gras, amyloid deposition was observed in several organs (54). Those findings raise concerns about the potential for diseases caused by amyloid accumulation, such as prion disease and Alzheimer’s disease, from consuming domestic duck organs. The transmission of diseases through the consumption of BSE-contaminated meat has already been confirmed as a cause of variant CJD. Therefore, further studies are needed to assess the susceptibility of domestic ducks to prion diseases and to consider the possibility that they could be susceptible species, unlike chickens.

## Data availability statement

The data presented in the study are deposited in the DRYAD repository (https://datadryad.org/stash/share/bijVKgL55WURNYXsXMfuos_ULtualWmKEBTTdJF8fuk).

## Ethics statement

The animal study was approved by Institutional Animal Care and Use Committee of Jeonbuk National University. The study was conducted in accordance with the local legislation and institutional requirements.

## Author contributions

M-JJ: Conceptualization, Formal analysis, Writing – original draft. ZW: Writing – review & editing. W-QZ: Writing – review & editing. Y-CK: Conceptualization, Formal analysis, Writing – review & editing. B-HJ: Conceptualization, Formal analysis, Writing – review & editing.

## Supplementary material

SUPPLEMENTARY FIGURE 1 Genotype and allele frequencies of prion protein gene (*PRNP*) polymorphisms in Pekin ducks. The bar graph illustrates the frequencies of genotype and allele of prion protein gene (*PRNP*) polymorphisms in Pekin ducks. The left graph represents genotype frequencies, and the right graph depicts allele frequencies. The numbers on the right side of the graph correspond to the values indicating the Hardy-Weinberg equilibrium (HWE).

SUPPLEMENTARY FIGURE 2 Haplotype frequencies of prion protein gene (*PRNP*) polymorphisms in Pekin ducks. The upper panel displays five distinct haplotypes derived from five prion protein gene (*PRNP*) polymorphisms in Pekin ducks, while the lower panel provides a graphical representation of the haplotype distribution. Asterisks (*) indicate nucleotide positions identical to ht1.

SUPPLEMENTARY FIGURE 3 Prediction of structural changes of non-synonymous single nucleotide polymorphisms (SNPs) in the Pekin duck prion protein gene (*PRNP*) on PrP. The tertiary structure illustrates the comparison of Pekin duck PrP with two different amino acids at residue 237, as analyzed by Missense3D. The tertiary structure modeling of Pekin duck PrP was constructed using Alphafold2. All chains are depicted in white, except for the proline of the wildtype amino acid (aqua) and the leucine of the variant amino acid (red) at residue 237.
