# Supplementary figures and images for: The first report of polymorphisms of the prion protein gene (PRNP) in Pekin ducks (Anas platyrhynchos domestica)
Source: Front Vet Sci. 2023 Nov 8;10:1273050. doi: 10.3389/fvets.2023.1273050 (PMC10664711; doi:10.3389/fvets.2023.1273050)

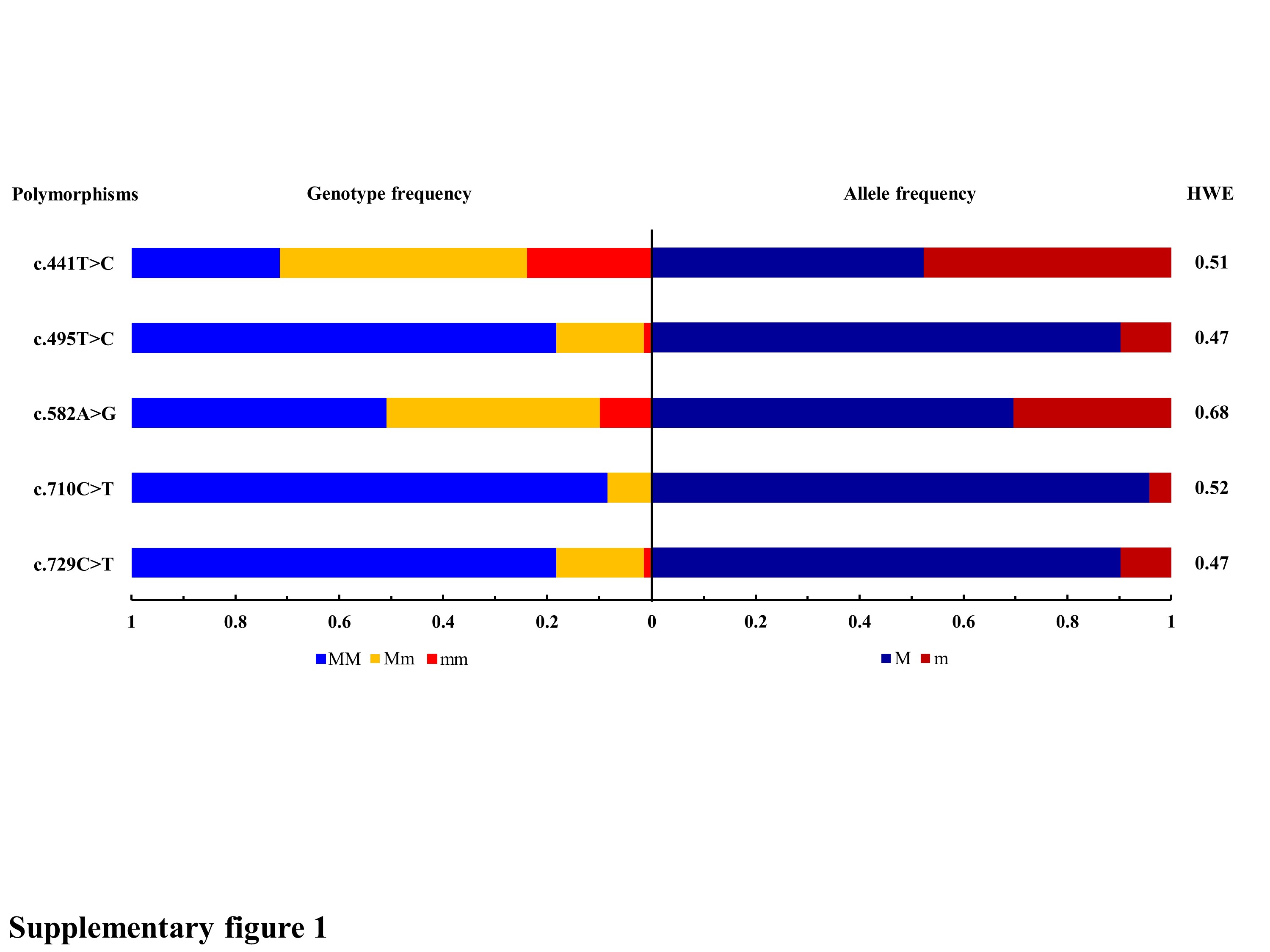

Supplement: SUPPLEMENTARY FIGURE 1 — Genotype and allele frequencies of prion protein gene (PRNP) polymorphisms in Pekin ducks. The bar graph illustrates the frequencies of genotype and allele of prion protein gene (PRNP) polymorphisms in Pekin ducks. The left graph represents genotype frequencies, and the right graph depicts allele frequencies. The numbers on the right side of the graph correspond to the values indicating the Hardy-Weinberg equilibrium (HWE). [file Image_1.JPEG]

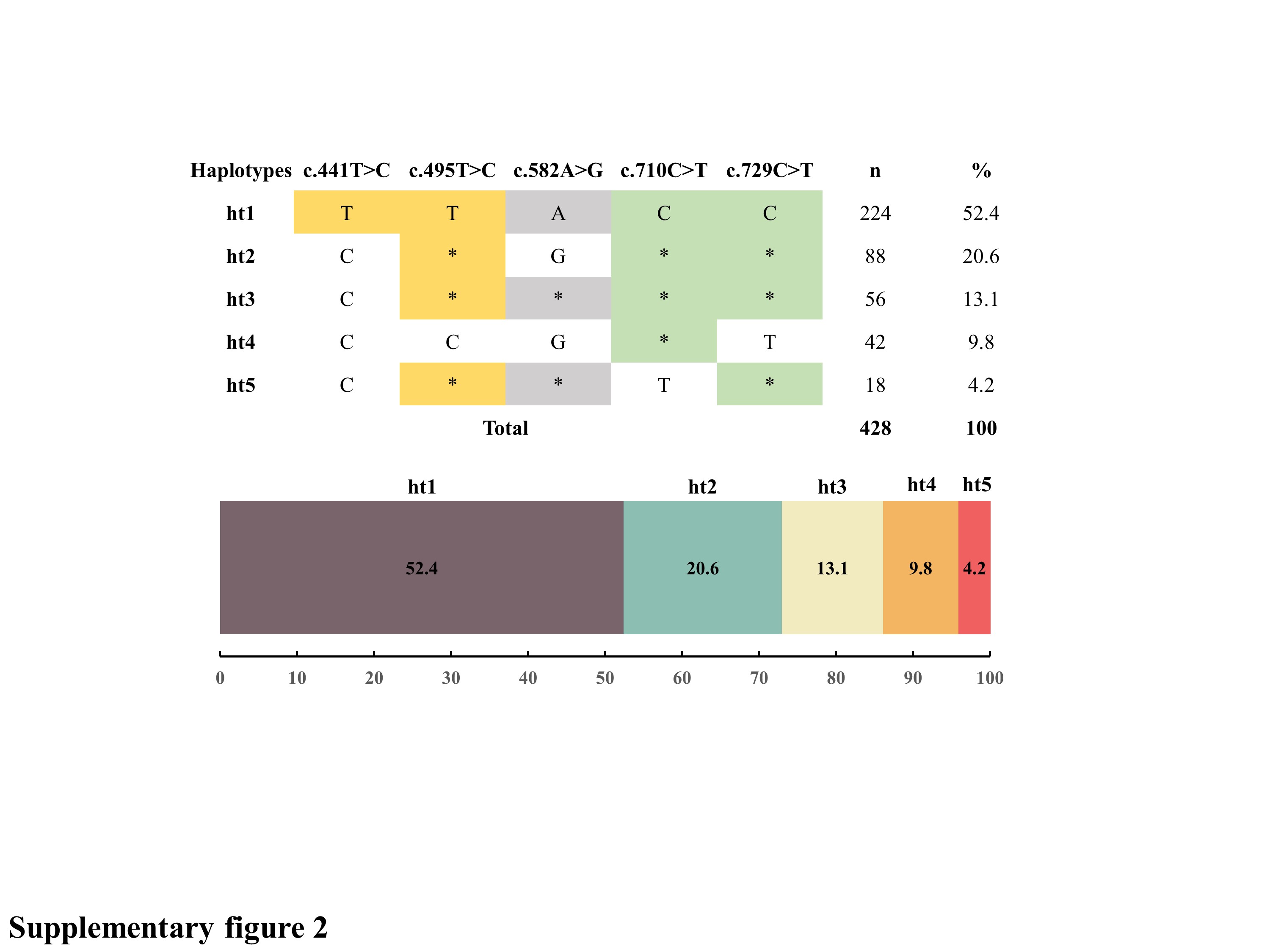

Supplement: SUPPLEMENTARY FIGURE 2 — Haplotype frequencies of prion protein gene (PRNP) polymorphisms in Pekin ducks. The upper panel displays five distinct haplotypes derived from five prion protein gene (PRNP) polymorphisms in Pekin ducks, while the lower panel provides a graphical representation of the haplotype distribution. Asterisks (*) indicate nucleotide positions identical to ht1. [file Image_2.JPEG]

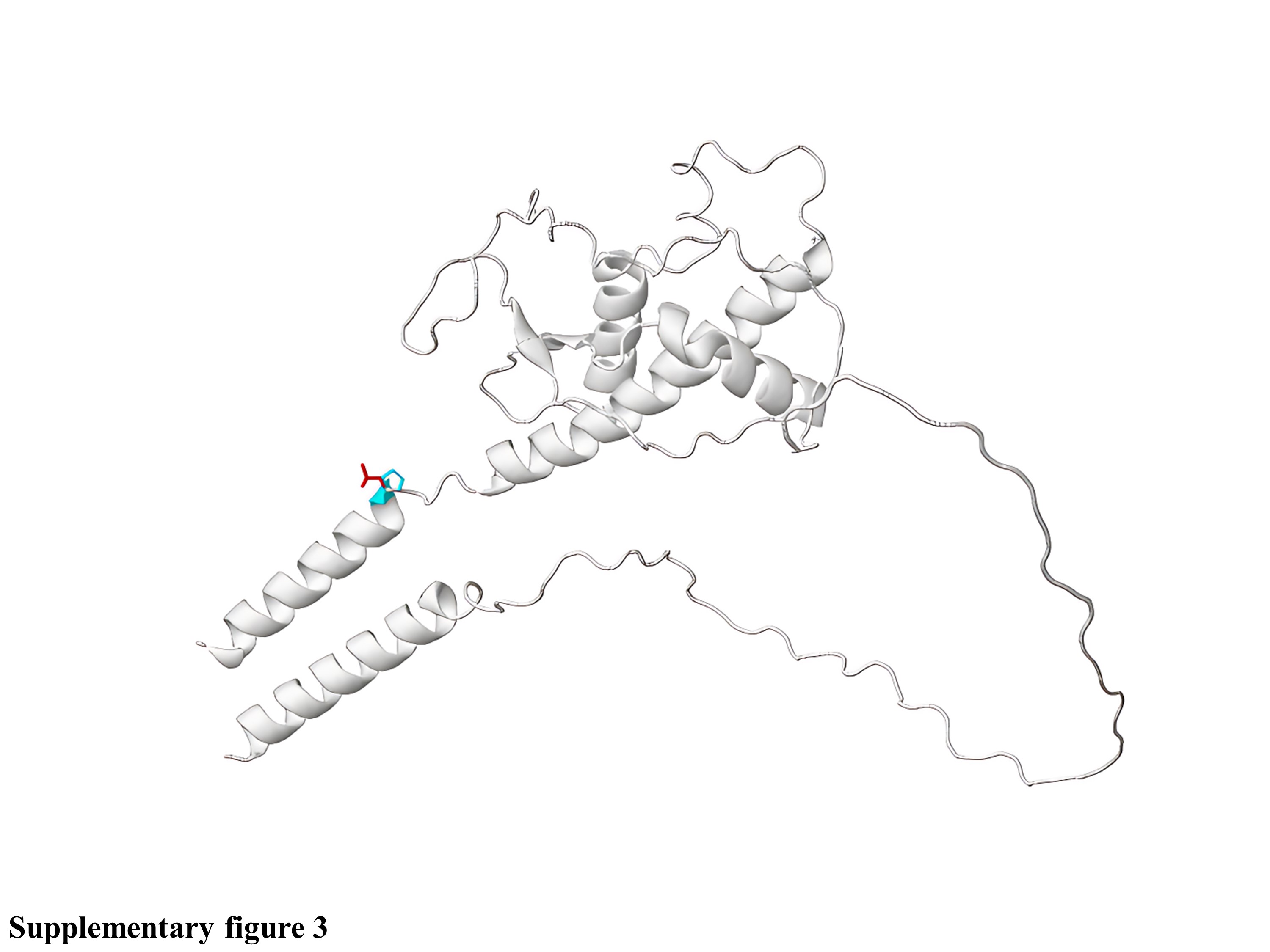

Supplement: SUPPLEMENTARY FIGURE 3 — Prediction of structural changes of non-synonymous single nucleotide polymorphisms (SNPs) in the Pekin duck prion protein gene (PRNP) on PrP. The tertiary structure illustrates the comparison of Pekin duck PrP with two different amino acids at residue 237, as analyzed by Missense3D. The tertiary structure modeling of Pekin duck PrP was constructed using Alphafold2. All chains are depicted in white, except for the proline of the wildtype amino acid (aqua) and the leucine of the variant amino acid (red) at residue 237. [file Image_3.JPEG]
